# Evaluating large language models for renal colic imaging recommendations: a comparative analysis of Gemini, copilot, and ChatGPT-4.0

**DOI:** 10.1186/s12245-025-00895-3

**Published:** 2025-07-04

**Authors:** Yavuz Yigit, Asım Enes Ozbek, Betul Dogru, Serkan Gunay, Baha AlKahlout

**Affiliations:** 1 Department of Emergency Medicine, Hamad Medical Corporation, Doha, 3050 Qatar; 2 Blizard Institute, Queen Mary University, London, UK; 3 Department of Emergency Medicine, Kocaeli City Hospital, Kocaeli, Turkey; 4 Department of Emergency Medicine, Hitit University, Çorum, Turkey; 5 College of Medicine, Qatar University, Doha, Qatar

**Keywords:** Large Language models (LLMs), Natural Language processing (NLP), Renal colic, Imaging recommendations, Gemini, Copilot, ChatGPT-4.0

## Abstract

**Background:**

The field of natural language processing (NLP) has evolved significantly since its inception in the 1950s, with large language models (LLMs) now playing a crucial role in addressing medical challenges.

**Objectives:**

This study evaluates the alignment of three prominent LLMs—Gemini, Copilot, and ChatGPT-4.0—with expert consensus on imaging recommendations for acute flank pain.

**Methods:**

A total of 29 clinical vignettes representing different combinations of age, sex, pregnancy status, likelihood of stone disease, and alternative diagnoses were posed to the three LLMs (Gemini, Copilot, and ChatGPT-4.0) between March and April 2024. Responses were compared to the consensus recommendations of a multispecialty panel. The primary outcome was the rate of LLM responses matching the majority consensus. Secondary outcomes included alignment with consensus-rated perfect (9/9) or excellent (8/9) responses and agreement with any of the nine panel members.

**Results:**

Gemini aligned with the majority consensus in 65.5% of cases, compared to 41.4% for both Copilot and ChatGPT-4.0. In scenarios rated as perfect or excellent by the consensus, Gemini showed 69.5% agreement, significantly higher than Copilot and ChatGPT-4.0, both at 43.4% (*p* = 0.045 and < 0.001, respectively). Overall, Gemini demonstrated an agreement rate of 82.7% with any of the nine reviewers, indicating superior capability in addressing complex medical inquiries.

**Conclusion:**

Gemini consistently outperformed Copilot and ChatGPT-4.0 in aligning with expert consensus, suggesting its potential as a reliable tool in clinical decision-making. Further research is needed to enhance the reliability and accuracy of LLMs and to address the ethical and legal challenges associated with their integration into healthcare systems.

**Supplementary Information:**

The online version contains supplementary material available at 10.1186/s12245-025-00895-3.

## Introduction

The field of natural language processing (NLP) has its origins in the 1950s, when early researchers laid the foundations for this interdisciplinary field at the intersection of linguistics, computer science, and artificial intelligence (AI). While the initial capabilities of NLP were limited and unsuitable for direct application in high-stakes medical decision-making, the field has since undergone a remarkable transformation. These large language models (LLMs) have been developed through significant advancements in AIand NLP, and have undergone extensive training on comprehensive datasets comprising scholarly publications, books, and guidelines. Since their public introduction, large language models (LLMs) have been employed in various scientific endeavors to address medical challenges, such as optimizing healthcare administration and tackling global health issues [1, 2]. These tools hold considerable potential for healthcare applications. Nonetheless, the research outcomes remain inconclusive due to several influencing factors, including the evaluation of different concepts, precision, and reproducibility [1, 3].

Urolithiasis-induced flank pain is a common reason for emergency department (ED) visits, with the incidence of these visits increasing significantly in recent years [4, 5]. Despite this rise, there remains uncertainty among ED clinicians regarding the optimal imaging modality for diagnosing obstructive kidney disorders. According to the latest guidelines from the European Association of Urology on urolithiasis, Non-contrast-enhanced CT (NCCT) is recommended for confirming the presence of stones in patients presenting with acute flank pain, following an initial ultrasound assessment [6]. NCCT is considered a valuable diagnostic tool, particularly in cases where the findings from the initial ultrasound examination are inconclusive. However, the guidelines lack clear guidance on specific clinical scenarios where CT scans may not be necessary for effectively managing patients with acute flank pain.

The consensus report by the American College of Emergency Medicine, the American College of Radiology, and the American Association of Urology asserts that CT imaging is often unnecessary in various clinical scenarios, and some patients may not require any imaging at all [7]. Despite these recommendations, a nationwide study of emergency department practices revealed a significant increase in the use of CT scans for patients presenting with suspected obstructive kidney disorders from 2012 to 2018 [8]. In contrast, the same study showed that ultrasonography was employed in fewer than 3% of ED visits involving suspected obstructive kidney disorders.

When the literature is examined, there is no study showing to what extent LLMs can be useful in choosing the right imaging method in renal colic patients. Also the association between LLMs and the consensus report, as well as their associated advantages, remains unknown. This study hypothesizes that, owing to their capacity to access extensive data, the three most prominent LLMs (OpenAI’s ChatGPT-4.0, Google’s Gemini, and Microsoft’s Copilot) could provide valuable insights into determining the necessity and appropriateness of imaging for patients presenting with acute flank pain in the emergency department. Specifically, this study aims to evaluate whether the recommendations of these three AI chatbots align with the consensus report when applied to 29 pre-defined clinical vignettes concerning imaging in cases of suspected renal colic.

## Methods

Since no patient or clinician data is used in the study, ethics committee approval is not required. The multispecialty consensus report by the American College of Emergency Medicine (ACEP), the American College of Radiology (ACR), and the American Association of Urology (AUA) provided twenty-nine distinct cases of acute flank pain, each representing different combinations of age, sex, pregnant status, likelihood of stone disease, and possibility of an alternative diagnosis [7]. The twenty-nine vignettes used in this study were derived from a consensus report developed by ACEP, ACR, and AUA, based on a systematic literature review and a structured modified Delphi process, ensuring that each vignette represented a clinically important and evidence-based case. Detailed information about the 29 vignettes is shown in Supplementary Table 1, which outlines the demographic and clinical characteristics included in each scenario.

Between March and April 2024, each vignette was presented with the same question pattern to the three LLMs, which include OpenAI’s ChatGPT-4.0, Google’s Gemini, and Microsoft’s Copilot. The same question was asked all of them ‘Please answer according to the up-to-date data, for this scenario which would be the most appropriate management method to perform in an ED: no (further) imaging or point-of-care ultrasonography or radiology-performed ultrasonography or reduced-radiation dose CT, standard CT (non-contrast) or CT with intravenous contrast.’ Responses generated by LLMs were documented on the digital data sheets. The agreement between the responses of LLMs was assessed. The level of consensus was determined as the number of expert panelists in agreement for each vignette, as defined by the original consensus report: perfect (9/9), excellent (8/9), good (6 to 7/9), moderate (5/9), and not reached (< 5/9). The answers covered a range of imaging choices, such as no (further) imaging, point-of-care ultrasonography, radiology-performed ultrasonography, reduced-radiation dose CT, standard CT (non-contrast), and CT with intravenous contrast. Furthermore, to ensure adherence to the consensus report, the replies were categorized into three main groups: no further imaging, ultrasonography, and CT. This grouping was prominent for consistency and comparability, as the LLM’s imaging recommendations were consistent with the predefined consensus responses used by the expert panel, facilitating analysis and interpretation of results. The responses of AI chatbots were compared both between themselves and with the responses of the consensus members. The reference study identified 9 consensus members, including representatives from 3 specialty societies: the American College of Emergency Physicians (ACEP), the American College of Radiology, and the American Urological Association. All consensus members were board-certified practicing physicians and were nominated based on their previous work on specialty-specific guidelines [7]. Each LLM’s answer was directly compared against the consensus response for each vignette, thereby ensuring alignment between the LLMs and the expert panel.

The primary outcome measure of the study was to compare the three LLMs for the rate of giving the same answers as the majority of the members of the consensus on previously defined 29 vignettes about imaging in suspected renal colic. The secondary outcome measures of the study were to determine the compatibility of three LLMs with these answers in the questions for which consensus was achieved at perfect and excellent level in the consensus report and to determine the agreement level of three LLMs with not the majority consensus, but by whether the answer of the 3 LLMs was consistent with the answer of any of the 9 members.

Statistical analyses were performed using Jamovi version 2.5.3.0 Computer Software. The agreement between the answers given by the LLMs and the consensus members was manually assessed separately in 3 groups (the majority of answers provided across all questions, the consensus ratings of “perfect” or “excellent.“, the response of any of the 9 reviewers that took part in the consensus). Results were given as number of correct answers and percentage. The agreement between the correct answers given by the LLMs was evaluated by the chi-square test in pairs, separately for each group. To statistically show the alignment of LLMs with a reference cohort, Fleiss’ kappa values have been calculated. P values less than 0.05 were considered statistically significant.

## Results

**Fig. 1:**
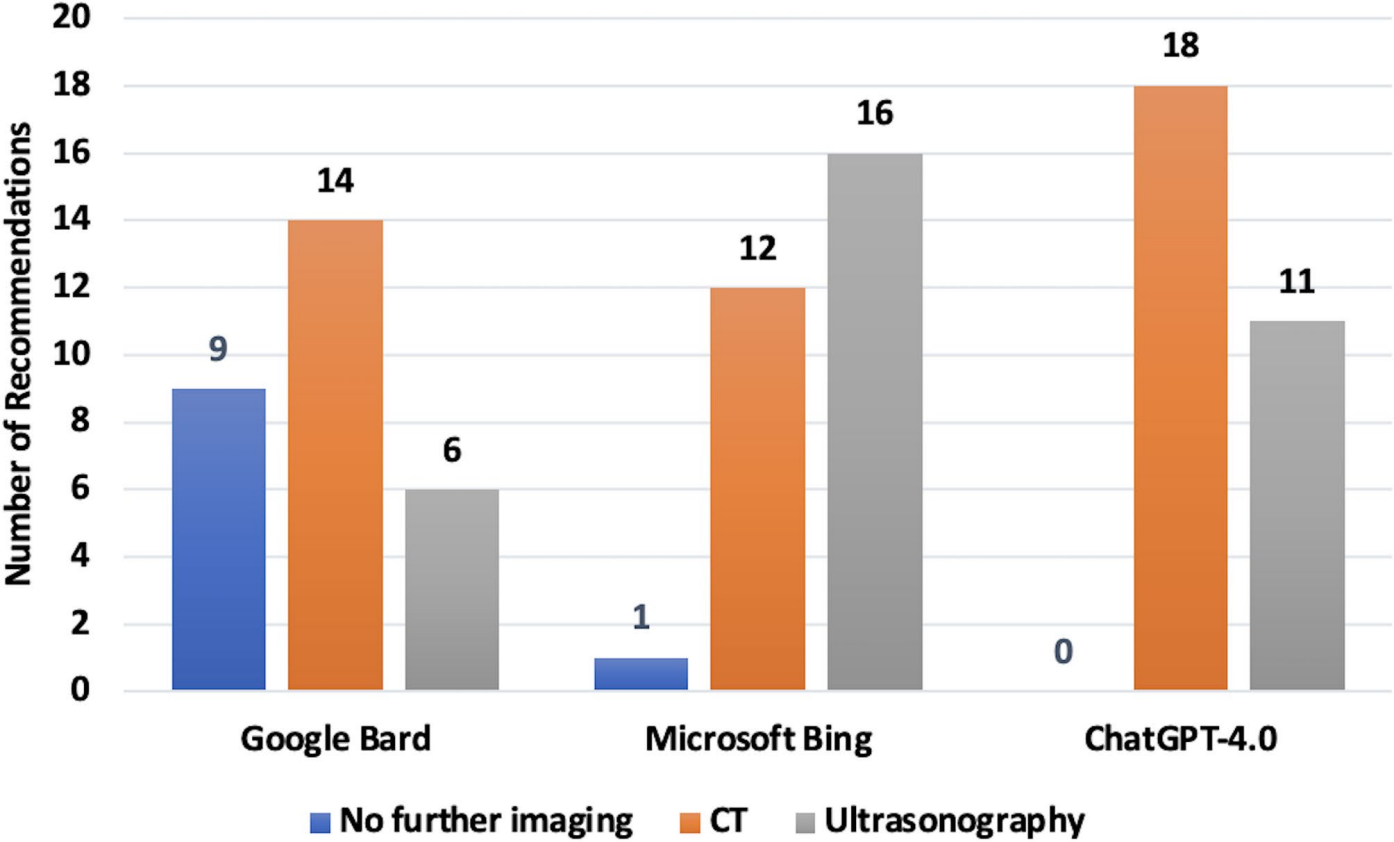
Number of recommendations

**Fig. 2:**
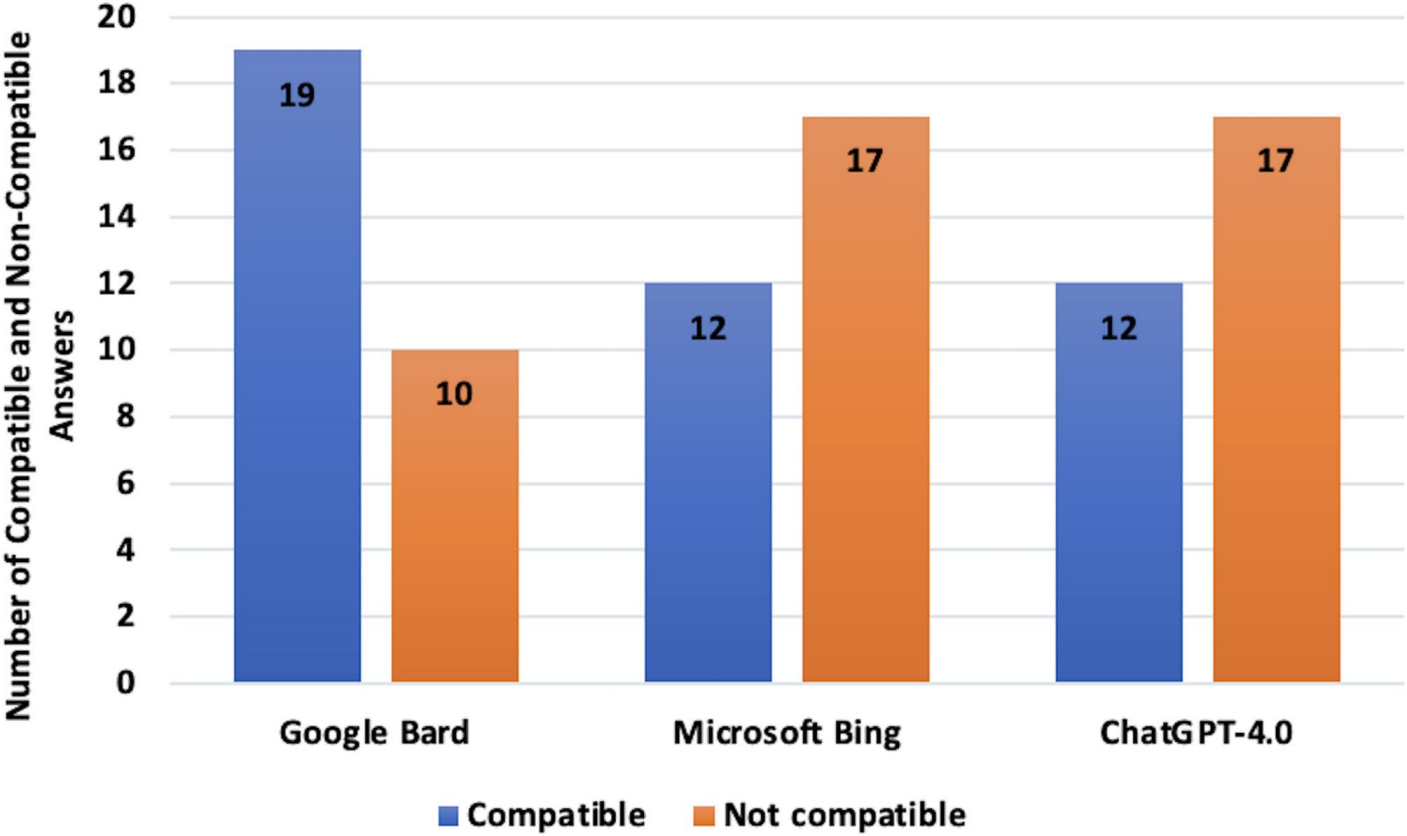
Number of compatible and non-compatible answers

Table 1 presents the individual responses of each LLM and their agreement with the priori consensus. Figure 1 illustrates the distribution of imaging recommendations (no imaging, CT, or ultrasonography) provided by each LLM. When evaluating the compatibility of the LLMs with the majority of answers provided across all questions, Gemini matched the consensus members’ responses in 19 questions (65.5%). In contrast, Copilot and ChatGPT-4.0 provided the same answers in 12 questions (41.4%) (Fig. 2).

**Table 1:** Answers and agreements of LLMs with the priori consensus responses

	Answers of LLMs			Agreement of LLMs^a^			Priori consensus degree^b^
Question No.	Gemini	Copilot	ChatGPT	Gemini	Copilot	ChatGPT	
1	RDCT	POCUS	NCCT	-	+	-	Moderate
2	POCUS	RDCT	NCCT	+	-	-	Moderate
3	RDCT	POCUS	RDCT	+	+	+	Good
4	RDCT	POCUS	NCCT	-	+	-	Perfect
5	RDCT	POCUS	NCCT	+	+	+	Excellent
6	RDCT	POCUS	RDCT	+	-	+	Perfect
7	RDCT	RDCT	NCCT	-	-	-	Perfect
8	No imaging	NCCT	NCCT	+	-	-	Perfect
9	RDCT	POCUS	NCCT	+	-	+	Perfect
10	RDCT	RDCT	NCCT	+	+	+	Excellent
11	No imaging	POCUS	NCCT	+	-	+	Excellent
12	RDCT	POCUS	NCCT	+	+	+	Excellent
13	RDCT	RDCT	NCCT	+	+	+	Good
14	RDCT	RDCT	NCCT	+	+	+	Excellent
15	No imaging	NCCT	NCCT	+	+	+	Moderate
16	RDCT	NCCT	NCCT	+	+	+	Excellent
17	RDCT	NCCT	NCCT	+	+	+	Perfect
18	RDCT	NCCT	RDCT	+	+	+	Perfect
19	POCUS	POCUS	RPUS	-	-	-	Perfect
20	No imaging	RPUS	RPUS	-	+	+	Perfect
21	No imaging	RDCT	RPUS	+	-	+	Excellent
22	POCUS	POCUS	RPUS	+	+	+	Perfect
23	No imaging	RDCT	RPUS	+	-	-	Perfect
24	No imaging	POCUS	POCUS	+	-	-	Perfect
25	POCUS	POCUS	POCUS	+	+	+	Perfect
26	No imaging	POCUS	RPUS	+	-	-	Perfect
27	No imaging	No imaging	RPUS	+	+	-	Excellent
28	POCUS	POCUS	POCUS	+	+	+	Good
29	POCUS	POCUS	RPUS	+	+	+	Perfect

+: Compatible, -: Not compatible RDCT: reduced-radiation dose computer tomography POCUS: point-of-care ultrasonography NCCT: non-contrast computer tomography RPUS: radiology-performed ultrasonography ^a^ the response of any of the 9 reviewers ^b^ According to a consensus report by the American College of Emergency Physicians, the American College of Radiology, and the American Urological Association; consensus was defined as perfect (9/9), excellent (8/9), good (6 to 7/9), moderate (5/9), and not reached (< 5/9) (7)

**Fig. 3:**
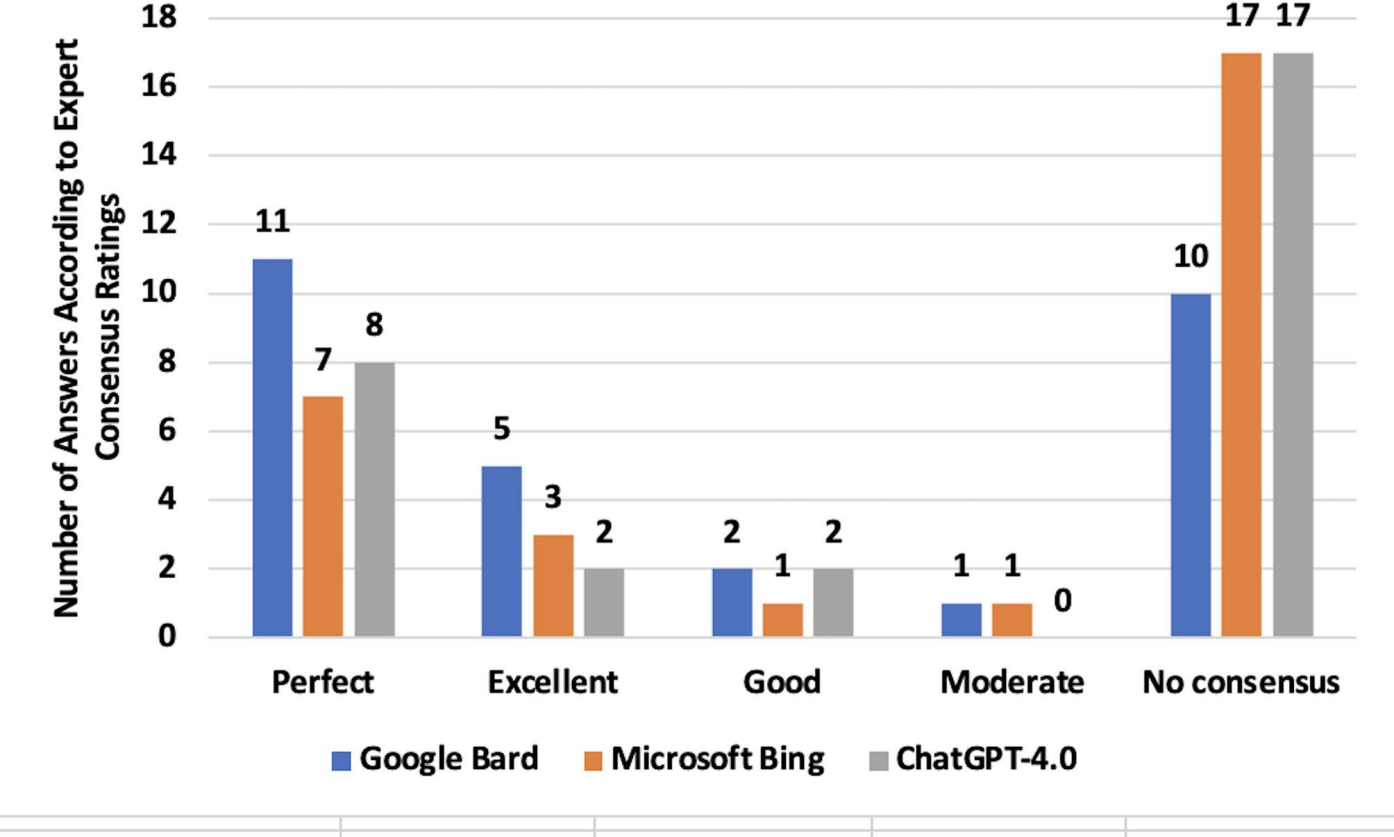
Number of answers according to expert consensus ratings

Gemini correctly answered 16 out of 23 questions (69.5%) where the consensus rated the answers as “perfect” or “excellent.” In contrast, both Copilot and ChatGPT-4.0 correctly answered 10 out of 23 questions (43.4%) in alignment with the consensus ratings of “perfect” or “excellent.” (Fig. 3). Gemini responses showed greater agreement compared to Copilot (*p* = 0.045) and ChatGPT-4.0 (*p* < 0.001), indicating a consistent advantage in performance. Furthermore, statistical analysis revealed a substantial difference in agreement between Copilot and ChatGPT-4.0 (*p* = 0.001).

**Fig. 4:**
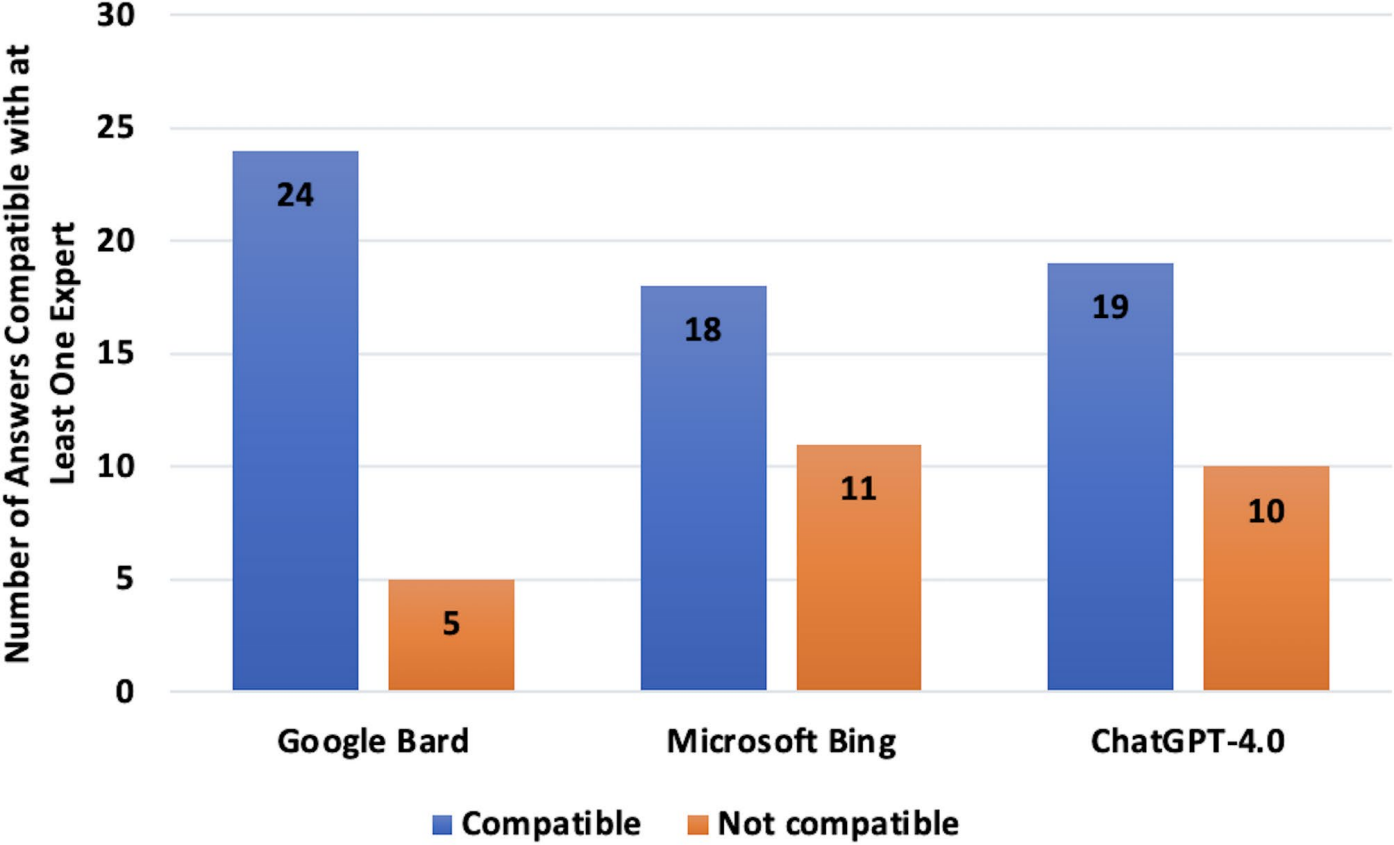
Number of answers compatible with at least one expert

Irrespective of the majority, Gemini had the highest level of agreement, with 82.7% (24/29), when evaluating the agreement of LLMs with the response of any of the 9 reviewers that took part in the consensus. Copilot responses agreement with 18 of the 29 clinical vignettes (62.1%), whereas ChatGPT-4.0 showed agreement in 19 cases (65.5%) (Fig. 4). No meaningful difference in agreement was observed between Gemini and Copilot (*p* = 0.917). In contrast, ChatGPT-4.0 demonstrated significantly different agreement rates compared to both Gemini (*p* = 0.019) and Copilot (*p* = 0.01). Fleiss’ kappa values for ChatGPT-4.0, Gemini, and Copilot in comparison with the nine expert reviewers were 0.638, 0.698, and 0.634, respectively—indicating substantial agreement for all three models.

## Discussion

In our study, we evaluated the alignment of three LLMs– Gemini, Copilot, and ChatGPT-4.0– with consensus answers provided by a panel of experts. The findings indicate that Gemini consistently demonstrated a higher degree of agreement with the expert consensus compared to the other LLMs examined. Specifically, Gemini excelled in compatibility with both the majority of the consensus members’ answers and with the responses of any of the nine reviewers who participated in the consensus, regardless of majority opinion. Additionally, among the three commonly utilized LLMs, Gemini was the most aligned in queries where a perfect or excellent consensus was achieved.

The extent to which LLMs can be utilized in clinical decision-making processes remains a topic of growing interest. To shed light on this issue, several studies have been conducted—and continue to be conducted—comparing the performance of various LLMs [9–12]. In a study involving 134 clinical cases, the diagnostic, therapeutic, and management-related decision-making accuracy of three different LLMs was evaluated, and Gemini was found to have the lowest overall performance [9]. In another study focused on surgical planning in glaucoma patients, Gemini demonstrated 32% lower agreement compared to ChatGPT-4, indicating inferior performance [10]. Similarly, when assessed as an intraoperative decision-support tool in plastic surgery, Gemini again exhibited suboptimal performance relative to ChatGPT-4 [11]. Conversely, another study evaluating the ability of ChatGPT-4 and Gemini to assess diagnosis and treatment plans for patients with acute cholecystitis found comparable performances between the two models [12]. In contrast to these previous studies, our study found that, in the context of selecting the appropriate imaging modality for patients with renal colic, Gemini produced responses that were more aligned with those of the expert consensus panel.

One potential factor contributing to variability in LLM responses is the influence of different guideline sources used during model training. While our study utilized the 2019 consensus report [7] as the reference standard, it is possible that the UK NICE guidelines, which have recommended low-dose non-contrast CT as the first-line imaging modality since January 2019, were included in the training datasets of the evaluated LLMs. This difference in guideline exposure may have contributed to discrepancies in LLM recommendations.

However, this does not undermine the validity of our findings, as our study was specifically designed to assess how well LLMs align with an established expert consensus rather than to evaluate the absolute correctness of their responses based on multiple guideline sources. In clinical practice, variations in recommendations across different guidelines are well-recognized and do not indicate an inherent flaw in an individual guideline or its interpretation. Future research could further investigate how different LLMs integrate and prioritize diverse clinical guidelines, providing additional insights into their decision-making processes.

Gemini demonstrated a significantly higher level of concurrence with the consensus, providing responses that were largely similar to those of the majority of consensus participants. This suggests that Gemini may have better performance in understanding and appropriately interpreting clinical case examples consistent with expert opinions. In contrast, only 41.4% of the questions were answered by ChatGPT-4.0 and Copilot in agreement with the consensus, indicating a less robust alignment with expert guidelines. Notably, Gemini achieved an agreement rate of 82.7% when comparing its overall responses with those of the nine reviewers. These findings indicate that Gemini could be a more credible tool for applications requiring strong conformity with expert guidelines [7]. In addition, Gemini’s high rate of agreement with expert evaluations suggests that it may assist clinicians in making imaging decisions in patients with renal colic.The high level of agreement of the Gemini scale with the highest scoring responses suggests that it may have the potential for widespread adoption in professional and academic contexts if supported by future studies. In addition, Gemini and other LLMs can have more sensitive evaluation capabilities with each new update. With the use of more data sets and the development of more fine analysis capabilities with each new update, more accurate results can be achieved in the clinical context. Future studies can contribute to the increase of our knowledge and experience in this subject by focusing on how the reliability and accuracy rates of these LLMs, especially Gemini, can be improved with new updates.

Although the use of AI-enabled LLMs may attract attention with their performance in clinical case assessments, the integration of AI technologies into healthcare systems raises significant ethical and legal concerns that warrant careful consideration [13, 14]. As these complex models become increasingly embedded in critical clinical decision-making processes, it is crucial to meticulously assess the multifaceted risks and responsibilities associated with their potential impact on patient outcomes. A primary and pressing ethical issue pertains to the transparency and explainability of the decision-making mechanisms within AI systems. Healthcare providers must be able to comprehend and trust the recommendations and rationale generated by these AI systems in order to maintain patient confidence and ensure appropriate treatment. The lack of explainability can lead to profound challenges in establishing clear lines of accountability, making it profoundly difficult to determine whether the healthcare professional or the AI system bears responsibility for an erroneous or suboptimal decision that could significantly impact a patient’s well-being [15]. This adaptation is essential for safeguarding patient trust and ensuring that the integration of AI into healthcare supports, rather than undermines, the quality and reliability of patient care.

Another concern regarding the usability of LLMs in clinical case evaluations is the legal processes related to the compliance of the use of AI in healthcare with standards regarding patient privacy and data protection [16]. In Turkiye, the Law on the Protection of Personal Data (KVKK) imposes strict regulations on the management and disclosure of patient data [17]. Similarly, in the United States, the Health Insurance Portability and Accountability Act (HIPAA) sets stringent rules on patient data handling [18]. AI systems must be designed to comply with these requirements in both jurisdictions, ensuring the safeguarding of patient information against unauthorized access and breaches. This highlights the importance of ensuring that AI systems in healthcare settings are designed to comply with stringent data protection regulations—such as KVKK in Türkiye and HIPAA in the U.S.—to safeguard patient information from unauthorized access and breaches. Legally, the utilization of AI in healthcare must adhere to standards pertaining to patient confidentiality and data protection [16]. The Health Insurance Portability and Accountability Act in the United States imposes strict rules on the management and disclosure of patient data [17]. AI systems must be engineered to adhere to these requirements, guaranteeing the safeguarding of patient information against unauthorized access and breaches.

### Limitations

This study has several limitations that should be considered when interpreting its findings. Firstly, the variability in the types and phrasing of questions posed to the LLMs can influence their responses, introducing variability that may affect the conclusions. However, the use of a standardized set of 29 clinical scenarios helps mitigate this variability, ensuring a consistent basis for comparison.

Secondly, the study’s generalizability is limited by the specific scenarios and questions presented. While the results may not apply to all medical inquiries, the selected scenarios are representative of common clinical situations in emergency departments. This relevance supports the applicability of the findings within the intended context. Thirdly, each vignette was presented only once in our study. Repetitive testing might increase the quality and robustness of the results of the study. Another limitation is that no power analysis was applied in our study. Instead, the study was designed using all vignettes in the consensus report. Finally, Copilot uses the infrastructure of ChatGPT-4.0. However, it also integrates the Microsoft database into its infrastructure, focusing on producing more balanced, creative and precise answers. Therefore, although they use similar infrastructures, they use different approaches to reach results on the given data, which distinguishes these two LLMs models from each other.

Despite these limitations, the study’s design and scenario selection provide a strong foundation for its conclusions. Future research can further address these limitations to enhance our understanding of LLMs in healthcare settings.

## Conclusion

This study demonstrates that Gemini exhibits a higher degree of alignment with expert consensus compared to Copilot and ChatGPT-4.0, has superior ability to address complex medical inquiries compared to other LLMs. Despite some limitations, the findings underscore the potential of Gemini to be a more reliable tool in clinical decision-making while also suggesting the need for further refinement and validation before its widespread adoption in clinical practice. Future research should focus on further improving the reliability and accuracy of LLMs while addressing ethical and legal challenges.

## Article summary

### Why is this topic important?

- The evaluation of large language models (LLMs) like Gemini, Copilot, and ChatGPT-4.0 is crucial as they have the potential to significantly influence clinical decision-making in healthcare.

### What does this study attempt to show?

- This study attempts to assess the alignment of responses from three prominent LLMs with expert consensus recommendations for imaging in acute flank pain scenarios.

### What are the key findings?

- Gemini demonstrated superior alignment with expert consensus, matching the majority consensus in 65.5% of cases, compared to 41.4% for both Copilot and ChatGPT-4.0.
- Gemini showed a higher agreement rate of 69.5% in scenarios rated as perfect or excellent by the consensus, significantly outperforming the other two LLMs.
- The overall agreement rate of Gemini with any of the nine reviewers was 82.7%, indicating its superior capability in addressing complex medical inquiries.

### How is patient care impacted?

- The use of Gemini can potentially enhance clinical decision-making by providing more reliable and expert-aligned recommendations.
- Integration of advanced LLMs like Gemini in emergency departments can improve diagnostic accuracy and patient management.

## Electronic supplementary material

Below is the link to the electronic supplementary material.

Supplementary Material 1

Supplementary Material 2

## Data Availability

The original data set will be availed by the corresponding author on reasonable request.
