# Supplementary material for: Evaluating large language models for renal colic imaging recommendations: a comparative analysis of Gemini, copilot, and ChatGPT-4.0
Source: Int J Emerg Med. 2025 Jul 4;18:123. doi: 10.1186/s12245-025-00895-3 (PMC12232162; doi:10.1186/s12245-025-00895-3)
Supplement: Supplementary file 1 — Supplementary Material 1 [file 12245_2025_895_MOESM1_ESM.docx]

Table 1: Answers and agreements of LLMs with the priori consensus responses

|  | Answers of LLMs | | | Agreement of LLMs^a^ | | | Priori consensus degree^b^ |
| --- | --- | --- | --- | --- | --- | --- | --- |
| Question No. | Gemini | Copilot | ChatGPT | Gemini | Copilot | ChatGPT |  |
| 1 | RDCT | POCUS | NCCT | - | + | - | Moderate |
| 2 | POCUS | RDCT | NCCT | + | - | - | Moderate |
| 3 | RDCT | POCUS | RDCT | + | + | + | Good |
| 4 | RDCT | POCUS | NCCT | - | + | - | Perfect |
| 5 | RDCT | POCUS | NCCT | + | + | + | Excellent |
| 6 | RDCT | POCUS | RDCT | + | - | + | Perfect |
| 7 | RDCT | RDCT | NCCT | - | - | - | Perfect |
| 8 | No imaging | NCCT | NCCT | + | - | - | Perfect |
| 9 | RDCT | POCUS | NCCT | + | - | + | Perfect |
| 10 | RDCT | RDCT | NCCT | + | + | + | Excellent |
| 11 | No imaging | POCUS | NCCT | + | - | + | Excellent |
| 12 | RDCT | POCUS | NCCT | + | + | + | Excellent |
| 13 | RDCT | RDCT | NCCT | + | + | + | Good |
| 14 | RDCT | RDCT | NCCT | + | + | + | Excellent |
| 15 | No imaging | NCCT | NCCT | + | + | + | Moderate |
| 16 | RDCT | NCCT | NCCT | + | + | + | Excellent |
| 17 | RDCT | NCCT | NCCT | + | + | + | Perfect |
| 18 | RDCT | NCCT | RDCT | + | + | + | Perfect |
| 19 | POCUS | POCUS | RPUS | - | - | - | Perfect |
| 20 | No imaging | RPUS | RPUS | - | + | + | Perfect |
| 21 | No imaging | RDCT | RPUS | + | - | + | Excellent |
| 22 | POCUS | POCUS | RPUS | + | + | + | Perfect |
| 23 | No imaging | RDCT | RPUS | + | - | - | Perfect |
| 24 | No imaging | POCUS | POCUS | + | - | - | Perfect |
| 25 | POCUS | POCUS | POCUS | + | + | + | Perfect |
| 26 | No imaging | POCUS | RPUS | + | - | - | Perfect |
| 27 | No imaging | No imaging | RPUS | + | + | - | Excellent |
| 28 | POCUS | POCUS | POCUS | + | + | + | Good |
| 29 | POCUS | POCUS | RPUS | + | + | + | Perfect |
| +: Compatible, -: Not compatible RDCT: reduced-radiation dose computer tomography POCUS: point-of-care ultrasonography NCCT: non-contrast computer tomography RPUS: radiology-performed ultrasonography ^a^ the response of any of the 9 reviewers ^b^ According to a consensus report by the American College of Emergency Physicians, the American College of Radiology, and the American Urological Association; consensus was defined as perfect (9/9), excellent (8/9), good (6 to 7/9), moderate (5/9), and not reached (<5/9) (7). | | | | | | | |
