# Supplementary material for: Evaluating large language models for renal colic imaging recommendations: a comparative analysis of Gemini, copilot, and ChatGPT-4.0
Source: Int J Emerg Med. 2025 Jul 4;18:123. doi: 10.1186/s12245-025-00895-3 (PMC12232162; doi:10.1186/s12245-025-00895-3)
Supplement: Supplementary file 2 — Supplementary Material 2 [file 12245_2025_895_MOESM2_ESM.docx]

**Appendix 1**. Clinical Vignettes, image responses from consensus members, and levels of agreement

| **Question No.** | **Vignettes** | **Agreement*** | **Answers** |
| --- | --- | --- | --- |
| 1 | A 35-y-old man with 2 previous kidney stones that passed spontaneously presents with an acute onset of flank pain during the last 3 h. He reports nausea with vomiting and has hematuria on urine dip. He has no abdominal tenderness. His pain is relieved after intravenous analgesics. | Moderate | 5 POCUS, 4 no imaging |
| 2 | A 55-y-old man with 2 previous kidney stones that passed spontaneously presents with an acute onset of flank pain during the last 3 h. He reports nausea with vomiting and has hematuria on urine dip. He has no abdominal tenderness. His pain is relieved after intravenous analgesics. | Moderate | 5 no imaging, 4 POCUS |
| 3 | A 75-y-old man with 2 previous kidney stones that passed spontaneously presents with an acute onset of flank pain during the last 3 h. He reports nausea with vomiting and has hematuria on urine dip. He has no abdominal tenderness. His pain is relieved after intravenous analgesics. | Good | 7 RDCT, 2 ultrasonography (1 POCUS, 1 RPUS) |
| 4 | A 35-y-old man with no history of kidney stones presents with an acute onset of flank pain during the last 3 h. He reports nausea with vomiting and has hematuria on urine dip. He has no abdominal tenderness. His pain is relieved after intravenous analgesics. | Perfect | 9 POCUS |
| 5 | A 55-y-old man with no history of kidney stones presents with an acute onset of flank pain during the last 3 h. He reports nausea with vomiting and has hematuria on urine dip. He has no abdominal tenderness. His pain is relieved after intravenous analgesics. | Excellent | 8 RDCT, 1 POCUS |
| 6 | A 75-y-old man with no history of kidney stones presents with an acute onset of flank pain during the last 3 h. He reports nausea with vomiting and has hematuria on urine dip. He has no abdominal tenderness. His pain is relieved after intravenous analgesics. | Perfect | 9 RDCT |
| 7 | A 35-y-old man with 2 previous kidney stones that passed spontaneously presents with an acute onset of flank pain during the last 3 h. He reports nausea with vomiting and has hematuria on urine dip. He has no abdominal tenderness. His pain is relieved after intravenous analgesics. Ultrasonography is performed; there is hydronephrosis on the side with the pain, and a stone is not visualized. | Perfect | 9 no imaging |
| 8 | A 35-y-old man with 2 previous kidney stones that passed spontaneously presents with an acute onset of flank pain during the last 3 h. He reports nausea with vomiting and has hematuria on urine dip. He has no abdominal tenderness. His pain is relieved after intravenous analgesics. Ultrasonography is performed; there is **no** hydronephrosis, and a stone is not visualized. | Perfect | 9 no imaging |
| 9 | A 35-y-old man with no history of kidney stones presents with an acute onset of flank pain during the last 3 h. He reports nausea with vomiting and has hematuria on urine dip. He has no abdominal tenderness. His pain is not relieved after intravenous analgesics. | Perfect | 9 RDCT |
| 10 | A 35-y-old man with no history of kidney stones presents with an acute onset of flank pain during the last 3 h. He reports nausea with vomiting and has hematuria on urine dip. He has no abdominal tenderness. His pain is relieved after intravenous analgesics. Ultrasonography is performed; there is hydronephrosis on the side with the pain, and a stone is not visualized. | Excellent | 8 no imaging, 1 RDCT |
| 11 | A 35-y-old man with no history of kidney stones presents with an acute onset of flank pain during the last 3 h. He reports nausea with vomiting and has hematuria on urine dip. He has no abdominal tenderness. His pain is relieved after intravenous analgesics. Ultrasonography is performed; there is **no** hydronephrosis, and a stone is not visualized. | Excellent | 8 no imaging, 1 RDCT |
| 12 | A 35-y-old woman with no history of kidney stones presents with an acute onset of flank pain during the last 3 h. She reports nausea with vomiting and has hematuria on urine dip. She has no abdominal tenderness. Her pain is relieved after intravenous analgesics. | Excellent | 8 ultrasonography (6 POCUS, 2 RPUS), 1 RDCT |
| 13 | A 35-y-old man with 2 previous kidney stones that passed spontaneously presents with left flank pain during the last 2 days. He reports nausea but no vomiting and has hematuria on urine dip. He has some left lower quadrant abdominal tenderness. His pain is relieved after intravenous analgesics. | Good | 7 POCUS, 2 RDCT |
| 14 | A 35-y-old man with 2 previous kidney stones that passed spontaneously presents with left flank pain during the last 2 days. He reports nausea but no vomiting and has hematuria on urine dip. He has some left lower quadrant abdominal tenderness. His pain is relieved after intravenous analgesics. Ultrasonography is performed; there is hydronephrosis on the side with the pain, and a stone is not visualized. | Excellent | 8 no imaging, 1 RDCT |
| 15 | A 35-y-old man with 2 previous kidney stones that passed spontaneously presents with left flank pain during the last 2 days. He reports nausea but no vomiting and has hematuria on urine dip. He has some left lower quadrant abdominal tenderness. His pain is relieved after intravenous analgesics. Ultrasonography is performed; there is **no** hydronephrosis, and a stone is not visualized. | Moderate | 5 no imaging, 4 RDCT |
| 16 | A 35-y-old man with no history of kidney stones presents with left flank pain during the last 2 days. He reports nausea but no vomiting and has hematuria on urine dip. He has some left lower quadrant abdominal tenderness. His pain is relieved after intravenous analgesics. | Excellent | 8 RDCT, 1 POCUS |
| 17 | A 55-y-old man with no history of kidney stones presents with left flank pain during the last 2 days. He reports nausea but no vomiting and has hematuria on urine dip. He has some left lower quadrant abdominal tenderness. His pain is relieved after intravenous analgesics. | Perfect | 9 CT (7 RDCT, 1 NCCT, 1 CT IV CON) |
| 18 | A 75-y-old man with no history of kidney stones presents with left flank pain during the last 2 days. He reports nausea but no vomiting and has hematuria on urine dip. He has some left lower quadrant abdominal tenderness. His pain is relieved after intravenous analgesics. | Perfect | 9 CT (5 NCCT, 4 CT IV CON) |
| 19 | A 35-y-old woman who is 10 weeks pregnant with no history of kidney stones presents with an acute onset of right flank pain during the last 3 h. She reports nausea with vomiting and has hematuria on urine dip. She has no abdominal tenderness. Her pain is relieved after intravenous analgesics. | Perfect | 9 ultrasonography (8 RPUS, 1 POCUS) |
| 20 | A 35-y-old woman who is 10 weeks pregnant with no history of kidney stones presents with an acute onset of right flank pain during the last 3 h. She reports nausea with vomiting and has hematuria on urine dip. She has no abdominal tenderness. Her pain is relieved after intravenous analgesics. Ultrasonography is performed; there is hydronephrosis on the side with the pain, and a stone is not visualized. | Perfect | 9 no imaging |
| 21 | A 35-y-old woman who is 10 weeks pregnant with no history of kidney stones presents with an acute onset of right flank pain during the last 3 h. She reports nausea with vomiting and has hematuria on urine dip. She has no abdominal tenderness. Her pain is relieved after intravenous analgesics. Ultrasonography is performed; there is **no** hydronephrosis, and a stone is not visualized. | Excellent | 8 no imaging, 1 RPUS |
| 22 | A 35-y-old woman who is 30 weeks pregnant with no history of kidney stones presents with an acute onset of right flank pain during the last 3 h. She reports nausea with vomiting and has hematuria on urine dip. She has no abdominal tenderness. Her pain is relieved after intravenous analgesics. | Perfect | 9 ultrasonography (8 RPUS, 1 POCUS) |
| 23 | A 35-y-old woman who is 30 weeks pregnant with no history of kidney stones presents with an acute onset of right flank pain during the last 3 h. She reports nausea with vomiting and has hematuria on urine dip. She has no abdominal tenderness. Her pain is relieved after intravenous analgesics. Ultrasonography is performed; there is hydronephrosis on the side with the pain, and a stone is not visualized. | Perfect | 9 no imaging |
| 24 | A 35-y-old man was treated in the ED the previous day with an acute onset of right flank pain, and a CT was performed that showed a 4-mm stone in the proximal right ureter, with some hydronephrosis. He presents today with recurrent, severe right flank pain. | Perfect | 9 no imaging |
| 25 | A 12-y-old boy with no history of kidney stones presents with an acute onset of flank pain during the last 3 h. He reports nausea with vomiting and has hematuria on urine dip. He has no abdominal tenderness. His pain is relieved after intravenous analgesics. | Perfect | 9 ultrasonography (7 RPUS, 2 POCUS) |
| 26 | A 12-y-old boy with no history of kidney stones presents with an acute onset of flank pain during the last 3 h. He reports nausea with vomiting and has hematuria on urine dip. He has no abdominal tenderness. His pain is relieved after intravenous analgesics. Ultrasonography is performed; there is hydronephrosis on the side with the pain, and a stone is not visualized. | Perfect | 9 no imaging |
| 27 | A 12-y-old boy with no history of kidney stones presents with an acute onset of flank pain during the last 3 h. He reports nausea with vomiting and has hematuria on urine dip. He has no abdominal tenderness. His pain is relieved after intravenous analgesics. Ultrasonography is performed; there is **no** hydronephrosis, and a stone is not visualized. | Excellent | 8 no imaging, 1 RDCT |
| 28 | A 35-y-old man with kidney stones who underwent shock-wave lithotripsy without stent placement 2 days ago presents with an acute onset of flank pain during the last 3 h. He reports nausea with vomiting and has hematuria on urine dip. He has no abdominal tenderness. His pain is relieved after intravenous analgesics. | Good | 7 ultrasonography (6 RPUS, 1 POCUS), 2 RDCT |
| 29 | A 35-y-old man with a 6-mm left-sided ureteral stone diagnosed by CT underwent stent placement yesterday. He presents with left flank and suprapubic pain worsening for the past 24 h. He has some nausea without vomiting. He has microscopic hematuria, but no abdominal tenderness. His pain is relieved after intravenous analgesics. | Perfect | 9 POCUS |

RDCT: reduced-radiation dose computer tomography POCUS: point-of-care ultrasonography NCCT: non-contrast computer tomography CT IV CON: intravenous contrast computer tomography RPUS: radiology-performed ultrasonography *According to a consensus report by the American College of Emergency Physicians, the American College of Radiology, and the American Urological Association; consensus was defined as perfect (9/9), excellent (8/9), good (6 to 7/9), moderate (5/9), and not reached (<5/9) (7).
